# Engineering responsive supramolecular biomaterials: Toward smart therapeutics

**DOI:** 10.1002/btm2.10031

**Published:** 2016-09-19

**Authors:** Matthew J. Webber

**Affiliations:** ^1^ Dept. of Chemical & Biomolecular Engineering University of Notre Dame Notre Dame IN 46556

**Keywords:** biomaterials, biomolecular engineering, drug delivery, supramolecular chemistry

## Abstract

Engineering materials using supramolecular principles enables generalizable and modular platforms that have tunable chemical, mechanical, and biological properties. Applying this bottom‐up, molecular engineering‐based approach to therapeutic design affords unmatched control of emergent properties and functionalities. In preparing responsive materials for biomedical applications, the dynamic character of typical supramolecular interactions facilitates systems that can more rapidly sense and respond to specific stimuli through a fundamental change in material properties or characteristics, as compared to cases where covalent bonds must be overcome. Several supramolecular motifs have been evaluated toward the preparation of “smart” materials capable of sensing and responding to stimuli. Triggers of interest in designing materials for therapeutic use include applied external fields, environmental changes, biological actuators, applied mechanical loading, and modulation of relative binding affinities. In addition, multistimuli‐responsive routes can be realized that capture combinations of triggers for increased functionality. In sum, supramolecular engineering offers a highly functional strategy to prepare responsive materials. Future development and refinement of these approaches will improve precision in material formation and responsiveness, seek dynamic reciprocity in interactions with living biological systems, and improve spatiotemporal sensing of disease for better therapeutic deployment.

## 1 Introduction

The dynamic physiological and biological features presented by disease inspire the development of creative therapeutic solutions with the ability to sense indicators of pathology and respond in a way that is in itself dynamic. As such, therapeutics designed using supramolecular principles could have broad impact.^1^, ^2^, ^3^ Supramolecular chemistry (i.e., “chemistry beyond the molecule”) is based on rational design of specific, directional, tunable, and reversible molecular recognition motifs that exploit dynamic noncovalent interactions to build highly organized systems across length scales.^4^, ^5^, ^6^, ^7^, ^8^ In some manifestations, additive or cooperative interactions can be leveraged to prepare supramolecular materials, with emergent properties that follow from the dynamic nature of their constituents.^9^, ^10^, ^11^, ^12^ For example, although a single noncovalent interaction may be weak in comparison to a covalent bond, the summation and directionality of many such interactions can result in materials having properties resembling those of traditional covalent‐crosslinked polymer networks.^13^ Additionally, a significant benefit to taking a supramolecular approach in engineering systems is derived from the dynamic nature of these noncovalent interactions, enabling a molecular‐level design approach to control properties in a manner that is reversible, highly tunable, and modular.^1^ As such, the supramolecular toolbox affords many strategies to prepare new therapeutics for the treatment of disease.

**Figure 1:**
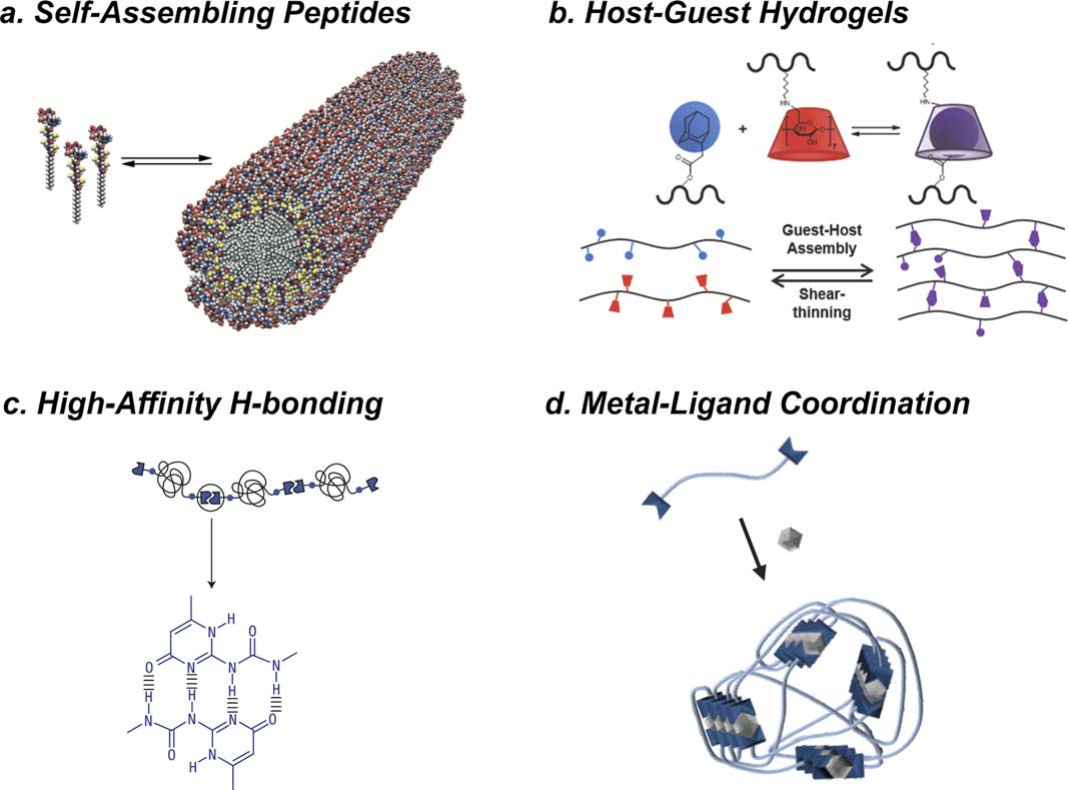
Examples of supramolecular materials that can be engineered with stimuli‐responsive properties, including (a) supramolecular assemblies of peptides based on long‐range order provided through β‐sheet‐like interactions, (b) polymeric materials cross‐linked via such supramolecular interactions as host‐guest complexation, (c) oligomeric precursors that can form extended chains through high‐affinity self‐complimentary hydrogen bonding units, such as the 2‐ureido‐pyrimidone motif, and (d) oligomeric precursors that form extended chains through end‐chain incorporation of metal‐chelating ligands. Panel (a) obtained from Hartgerink et al.^8^ and reprinted with permission from AAAS. Panel (b) reprinted with permission from Rodell et al.^45^ Panel (c) reprinted with permission from Macmillan Publishers Ltd: Nature Materials (Dankers et al.^51^), Copyright 2005. Panel (d) reprinted with permission from Macmillan Publishers Ltd: Nature (Burnworth et al.^62^), Copyright 2011

Several classes of supramolecular materials have been used toward applications relevant for therapy (Figure 1).^1^ Of these, supramolecular peptide assemblies have been extensively studied.^14^, ^15^, ^16^, ^17^, ^18^, ^19^, ^20^, ^21^, ^22^, ^23^, ^24^, ^25^, ^26^, ^27^, ^28^ Most often, supramolecular peptide assemblies arise from stackable motifs, typically β‐sheet‐like amyloid segments, that lead to high aspect‐ratio nanostructures with a stacking axis parallel to the length of the filamentous assembly.^8^, ^29^, ^30^, ^31^, ^32^, ^33^, ^34^, ^35^, ^36^ Another supramolecular motif, leveraging a macrocyclic “host” with a portal selective for inclusion of certain “guest” molecules, forms the basis for a number of supramolecular materials, owing to the typically high affinity and specificity of these interactions.^37^, ^38^, ^39^, ^40^, ^41^, ^42^, ^43^, ^44^, ^45^ Although hydrophobic interactions serve as the primary driving force for most host‐guest binding in aqueous environments, certain macrocyclic hosts combine hydrophobic interactions with hydrogen bonding between the host and guest, leading to affinities for some host‐guest pairs stronger even than that displayed by biotin‐avidin.^46^, ^47^, ^48^ Supramolecular polymers prepared using complimentary or self‐complimentary hydrogen bonding motifs to facilitate either chain‐extension or interchain crosslinks of oligomeric precursors have also been widely used in the context of therapeutic applications.^49^, ^50^, ^51^, ^52^, ^53^, ^54^, ^55^ In a similar way, metal‐ligand coordination chemistry can be used to prepare polymeric materials where a metal ion serves to promote crosslinking or chain extension of ligand‐bearing oligomers.^56^, ^57^, ^58^, ^59^, ^60^, ^61^, ^62^, ^63^ In all cases, each supramolecular motif has a characteristic equilibrium association constant (*K*_eq_), as well as specific binding kinetics defined by their rate constants of association (*k*_a_) and dissociation (*k*_d_), although this can be an overly simplistic approximation for multivalent or multimodal association motifs. Moreover, many supramolecular materials explore a complex free energy space, and the particular assembly pathway or environmental conditions can promote the existence of nonequilibrium and/or metastable conformations.^64^ As such, both the thermodynamics and kinetics of a supramolecular system contribute to the dynamic nature and responsiveness of these systems, and should be accounted for in molecular design and material processing.

There are many examples demonstrating the utility of supramolecular engineering in the context of preparing biomaterials, drug delivery devices, and scaffolds for tissue engineering, and these have been reviewed elsewhere.^1^ The focus here is to expand specifically on the methods used to prepare supramolecular systems that are responsive, that is can undergo structural or property changes elicited by some mechanism of control, specifically focusing on strategies relevant for use in engineering therapeutics and devices for biological sensing and disease diagnostics. It is furthermore important to note that this review will focus specifically on supramolecular approaches to the design of materials in pursuit of responsive therapeutics. As such, this contribution will highlight systems that use rationally designed supramolecular motifs as their mechanism for organization and/or function. This will necessarily exclude from discussion systems that arise through less specific or organized mechanisms of self‐assembly based on hydrophobic effects or electrostatic interactions (e.g., liposomes, polymersomes, polyplexes, nano‐networks, etc.), and these have been reviewed extensively elsewhere.^65^, ^66^, ^67^, ^68^, ^69^, ^70^

## 2 Preparing responsive supramolecular materials

There have been extensive efforts to prepare responsive supramolecular materials.^71^ The ability to precisely control the assembly state, structure, bioactivity, and mechanical properties using a variety of biologically relevant or biologically compatible triggers has obvious applications for more precise control of therapeutics. In some cases, these triggering events induce reversible changes in supramolecular materials, while in other cases they necessitate degradative processes that are inherently irreversible. For certain applications, reversible properties such as self‐healing mechanics or “on/off” switches may be desirable, but for others a sudden irreversible change in material properties may be more appropriate in the context of a “burst” response.

### 2.1 Externally applied stimuli

**Figure 2:**
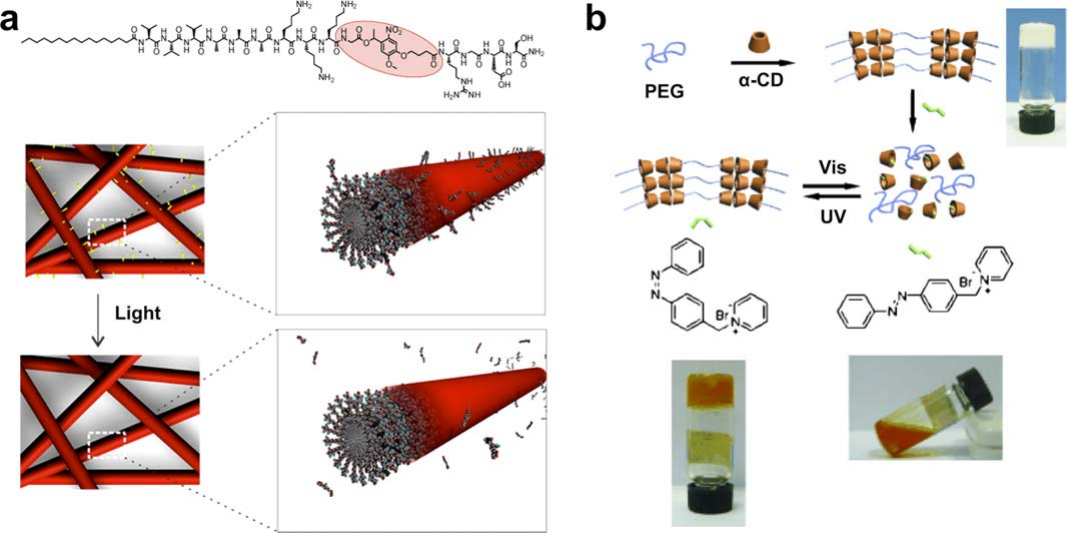
Examples of light‐responsive supramolecular materials. (a) Supramolecular peptide assemblies can be prepared from molecules that contain a cleavable nitrobenzyl group in their backbone, whereby upon light‐mediated cleavage a presented biologically active signal to promote cell adhesion is released from the surface of the nanofiber. (b) Threading of α‐cyclodextrin (α‐CD) onto polyethylene glycol (PEG) promotes gelation. A competitive guest containing a photoswitchable *trans‐*azobenzene can be added to competitively bind to and displace the α‐CD, leading to a *sol* that upon irradiation with UV light forms the nonbinding *cis*‐azobenzene, restoring the gel. The *sol‐gel* transition is reversible by using visible light to convert the guest back to its *trans* conformation. Panel (a) reprinted with permission from Allen and Cullis,^78^ Copyright 2012 American Chemical Society. Panel (b) reprinted with permission from Liao et al.^83^

Externally applied triggers such as light, heat, or ultrasound, along with electric or magnetic fields, have been common in the development of triggered drug delivery systems.^72^, ^73^, ^74^, ^75^ Supramolecular materials have similarly been developed to respond to externally applied triggers, and undergo a concomitant change in conformation, nanostructure, or bulk properties as a result. Light, specifically at ultraviolet wavelengths, has been perhaps the most used of these external triggers for responsive supramolecular systems. For example, the incorporation of a common photoresponsive group, the nitrobenzyl moiety, within supramolecular peptide building blocks was shown to enable external control over the geometry, shape, and interfacial curvature of the resulting assembly, and was also used to facilitate light‐triggered changes in bioactivity or assembly state of the material (Figure 2a).^76^, ^77^, ^78^, ^79^ The same nitrobenzyl chemistry can alternatively be used to cage a common supramolecular motif, 2‐ureido‐pyrimidone (UPy), for light‐triggered supramolecular polymerization.^80^, ^81^ Another common light‐sensitive chemistry used to prepare responsive supramolecular materials is the azobenzene‐type chemistry, which undergoes a reversible *cis‐trans* conformational change with cycling of irradiation between ultraviolet and visible light.^82^ Photo‐switching azobenzene guests bind to a macrocyclic cyclodextrin host in their *trans* conformation but do not bind in their *cis* conformation, enabling the creation of supramolecular hydrogels that can undergo a reversible *sol‐gel* transition with applications for use in light‐triggered release of drugs or proteins (Figure 2b).^83^, ^84^, ^85^, ^86^ In an interesting application, applied light absorbed by conjugated metal‐ligand motifs can produce heat that results in reversible dissociation of the metal‐ligand complex and concomitant reversible changes in material properties.^62^ In other cases, secondary photo‐crosslinking can be used to stabilize materials templated from supramolecular interactions, for example, by using diacetylene to induce crosslinking among self‐assembling monomers,^87^, ^88^, ^89^, ^90^ methacrylate chemistry to crosslink supramolecular protein assemblies,^91^ or thiol‐ene chemistry to crosslink the oligomeric backbones of metallo‐supramolecular polymers.^92^ Light presents a facile route to change or modify the structure or properties of a supramolecular material, and this approach could be further integrated with advances in two‐photon techniques for use with visible wavelengths and enhanced spatial control of the stimuli. In the context of therapeutic applications, however, the modest penetration depth of light within tissue could prove limiting to its ultimate use in externally controlled, minimally invasive therapies.

Modulation of temperature is another common trigger to control material properties. Especially in the case of supramolecular materials, applied thermal energy can enable myriad changes to material properties, acting as a dissipative force, promoting conformational changes that result in changes to supramolecular interactions, or enabling an assembly to overcome an energy barrier in accessing a new space within its free energy landscape.^93^ In the context of therapy, a distinction must be made between thermally responsive systems that function only under applied heat (i.e., temperature elevation far‐exceeding the physiologic norm) and those that undergo a change in properties in response to physiologically relevant changes in environmental temperature (discussed in the next section). To access temperatures required to prompt a phase change in certain supramolecular materials may still be achievable through adaptation of laser methodology used in localized thermal ablation, where temperatures in small regions of tissue can be controlled within the range of 50–100°C as part of increasingly routine medical practice.^94^ Given this expanded temperature landscape far beyond typical temperatures encountered physiologically, many strategies to prepare thermally responsive supramolecular materials could have relevance.^71^ Supramolecular polymers prepared from chain‐extension with UPy motifs, for example, exhibit properties resembling those of thermoplastic elastomers that enable their reversible processing and molding using applied heat.^51^, ^95^ Applied heat can also be used as a stimulus to disrupt and displace host‐guest complexes used in the crosslinking of supramolecular materials, leading to *sol‐gel* transitions that are fully reversible to restore mechanical integrity of the hydrogel following cooling.^43^, ^96^

While light and temperature have been more extensively used external stimuli to control the properties of supramolecular materials, magnetic fields have been leveraged by combining magnetically active inorganic nanoparticles, such as iron oxide, within supramolecular materials, enabling magnetically‐induced drug release^97^ or linear alignment of supramolecular assemblies.^98^ Similarly, electric fields have also been used to induce alignment in supramolecular assemblies with intrinsically large dipole moments.^99^ Mixed‐stack supramolecular assemblies form dipoles where polarization can be switched through application of an external electric field,^100^ while rotaxane assemblies can create molecular machines controlled through an external electric field.^101^ Ultrasound has also been used as a dissipative force or thermodynamic accelerator in controlling supramolecular structures. For example, ultrasound can be used to overcome kinetic barriers and enable reversible reconfiguration of a peptide nanostructure,^102^ promote disassembly of supramolecular micellar structures,^103^ or induce reversible polymerization or gelation in a supramolecular material.^104^, ^105^, ^106^ The clinical use of high‐intensity focused ultrasound suggests that ultrasound‐mediated control of supramolecular materials could have broad relevance for therapeutic application, and this approach certainly warrants further consideration in designing “smart” therapies.

### 2.2 Environmental modulation

**Figure 3:**
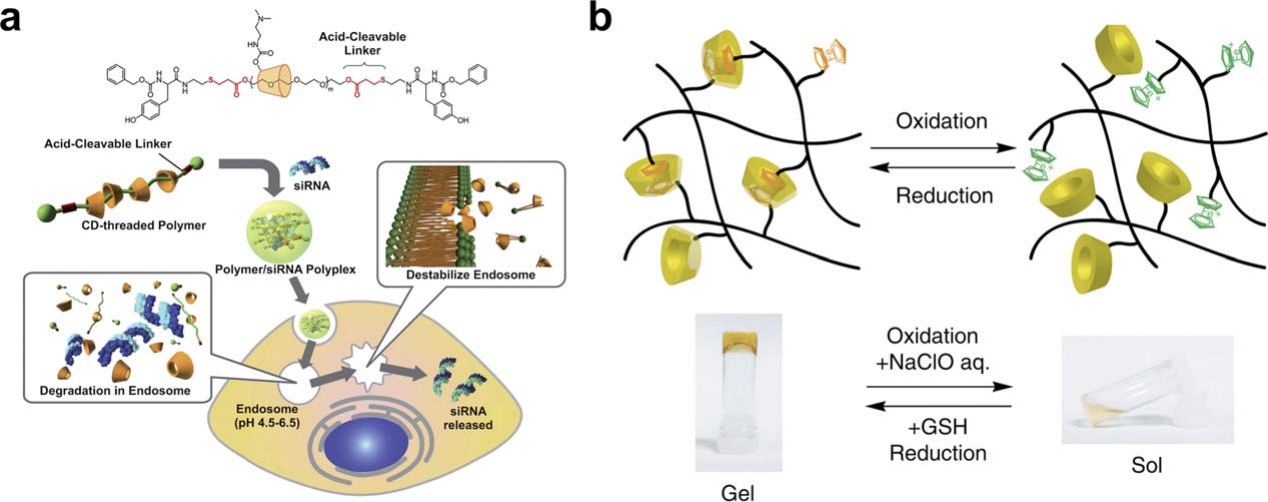
Examples of environmentally controlled supramolecular materials. (a) An acid‐sensitive polyrotaxane demonstrated for use in complexing with and delivering siRNA, whereby the acid‐labile capping ends are released during acidification in the endosome leading to release of α‐CD which destabilizes the endosomal membrane and promotes delivery of siRNA into the cytosol. (b) Redox‐responsive supramolecular gelation whereby the oxidation state of a pendant ferrocene guest can be used as a trigger to facilitate a reversible *sol‐gel* transition through altering the guest binding affinity to a pendant cyclodextrin host. Panel (a) reprinted from Tamura and Yui^115^ with permission from the Royal Society of Chemistry. Panel (b) reprinted with permission from Macmillan Publishers Ltd: Nature Communications (Nakahata et al.^135^), Copyright 2011

Local environmental changes, including pH, ionic strength, temperature, and redox potential, are also common triggers used for stimuli‐responsive drug delivery systems.^69^, ^107^, ^108^, ^109^ These have likewise been used to prompt changes in supramolecular materials. Supramolecular association often results from molecules that undergo a transition from a soluble monomeric form to a gelator as pH is transitioned across the p*K*_a_ of chargeable groups in the molecule and solubility and/or electrostatic repulsion are reduced. For example, supramolecular peptide assemblies must overcome electrostatic repulsion from charged amino acid side‐chains to stack and assemble, and the charge density of these residues is a direct function of the environmental pH, enabling *sol‐gel* transitions as pH is changed that alter the release of an encapsulated payload from a hydrogel network.^35^, ^110^, ^111^ The dependence of supramolecular interactions on pH also affords opportunities for pH‐triggered release of encapsulated cargo, such as a drug, when the material encounters a change in pH.^112^, ^113^, ^114^, ^115^, ^116^ Changing pH can alter the affinity for supramolecular host‐guest complexes by changing the protonation state of either the host or guest.^96^ Supramolecular host‐guest interactions can also be used as a “stoppering” valve to trap a soluble payload within a porous nanoparticle, where pH‐mediated displacement of the macrocyclic host results in release of the soluble drug from within the porous particle.^117^, ^118^, ^119^ Changes in pH have relevance in normal physiology and disease, as seen during acidification within an endosome (Figure 3a),^120^ fluctuation at various points in the digestive tract,^121^ and the slightly acidic character of the extracellular milieu as a part of cancer pathology.^122^

Based on similar principles to those governing pH‐responsiveness, increasing the ionic strength of a solution through the addition of salts can also be used to trigger the assembly of a supramolecular material by reducing the Debye screening length and decreasing electrostatic repulsion between charged residues. This sensitivity to salt provides an assembly pathway whereby different kinetically trapped nanostructures can be accessed.^64^ The presence of divalent ions can also be used to trigger the assembly of a small molecule by facilitating ionic bridging between nearby charged groups.^123^, ^124^ Supramolecular polymers designed to undergo chain extension and/or crosslinking through metal‐ligand coordination bonds are, by design, responsive the presence of specific salts in solution and often undergo *sol‐gel* transitions or dramatic increases in viscosity upon addition of appropriate metal ions.^60^, ^125^ Unlike pH which can vary among different compartments in the body or cell, the ionic strength in a physiologic setting remains more constant, and thus the opportunities for physiologic sensing using modulation of ionic strength are more limited. That said, changes that arise immediately upon injecting or implanting supramolecular materials are relevant in designing therapy, as this can allow a *sol* to be delivered through minimally invasive means (e.g., a syringe or catheter) in a deionized carrier, and then subsequently assemble into a material in the body upon the encountered elevation in ionic strength.

The inherent temperature transition that occurs between ambient and physiologic conditions can also be used to design responsive supramolecular materials. As stated in the previous section, for therapeutic applications a distinction is made here between temperatures that are realistically encountered in the body (i.e., 37°C) and much higher temperatures that can be achieved from applied heating. In peptide self‐assembly, intramolecular folding of an oligomer into a gel‐forming unit can be tuned through amino acid selection such that a dramatic *sol‐gel* phase change can be realized in physiologically relevant temperatures.^126^ Peptide sequences derived from thermally responsive elastin‐like peptides, which undergo an inverse temperature transition from hydrophobic to hydrophilic character, can be tuned within a physiologically relevant range to enable thermal sensing in materials.^127^, ^128^ Although such hydrophobic interactions do not, in and of themselves, constitute supramolecular interactions, the use of such a hydrophobic phase change in combination with other supramolecular motifs could have broad application in deriving thermally responsive supramolecular materials. For example, supramolecular protein‐derived materials based on receptor‐ligand interactions can be combined with a thermoresponsive polymer that undergoes a inverse temperature transition, poly(*N*‐isopropylacrylamide), to facilitate double‐network formation in situ following injection into physiologic temperatures and serves as a reinforcing network for the underlying supramolecular network in increasing the modulus and durability of the hydrogel.^129^ Like strategies that rely on ionic strength, these types of temperature‐responsive transitions would be inherently useful in developing materials that could be injected or implanted through minimally invasive means.

The generation of materials that are sensitive to redox (reduction/oxidation) reactions that occur in certain physiologic scenarios have also demonstrated promise in preparing “smart” materials for drug delivery.^130^ Similarly, supramolecular materials can be designed with redox‐responsive elements, such as by incorporation of reducible di‐sulfide linkages into the backbone of a supramolecular oligomer that rupture in response to redox conditions and compromise material properties.^131^ Reversible redox reactivity can also be used to tune the binding affinity for a complimentary supramolecular hydrogen bonding motif.^132^ Similarly, in metal‐ligand interactions the affinity of the ligand for metal ions can be altered reversibly as a function of redox conditions, enabling *sol‐gel* transitions coupled with changes in electrochromic properties as a function of redox state.^133^ Oxidation of the metal center in metal‐ligand supramolecular motifs can also be used to trigger structural changes in a material.^134^ Host‐guest interactions between ferrocene and cyclodextrin are also reversibly responsive to redox conditions which affect the state of the ferrocene guest, leading to complex formation under reducing conditions and decoupling under oxidative conditions, and can be used as a redox‐responsive crosslinking strategy to prepare materials when the host and guest are appended to a polymeric building block (Figure 3b).^135^ Redox‐switchable bistable rotaxanes, whereby two tethered guests have different affinities for a macrocycle as a function of redox conditions, can also be used to prepare nanovalves on mesoporous silica for triggered delivery of encapsulated payload.^136^ In the context of therapeutic applications, there are a number of biological redox signals that could be used in designing specific triggers for responsive supramolecular materials. These include reactive oxygen species and biologically relevant antioxidants such as glutathione which have implications in both health and disease,^137^ and their imbalance in the latter could serve as a trigger to generate responsive supramolecular materials. For example, in preparing a supramolecular pro‐drug assembly, the drug can be tethered via a reducible disulfide, for dissociation of the supramolecular carrier and drug release in the presence of glutathione.^138^

### 2.3 Biologically regulated materials

**Figure 4:**
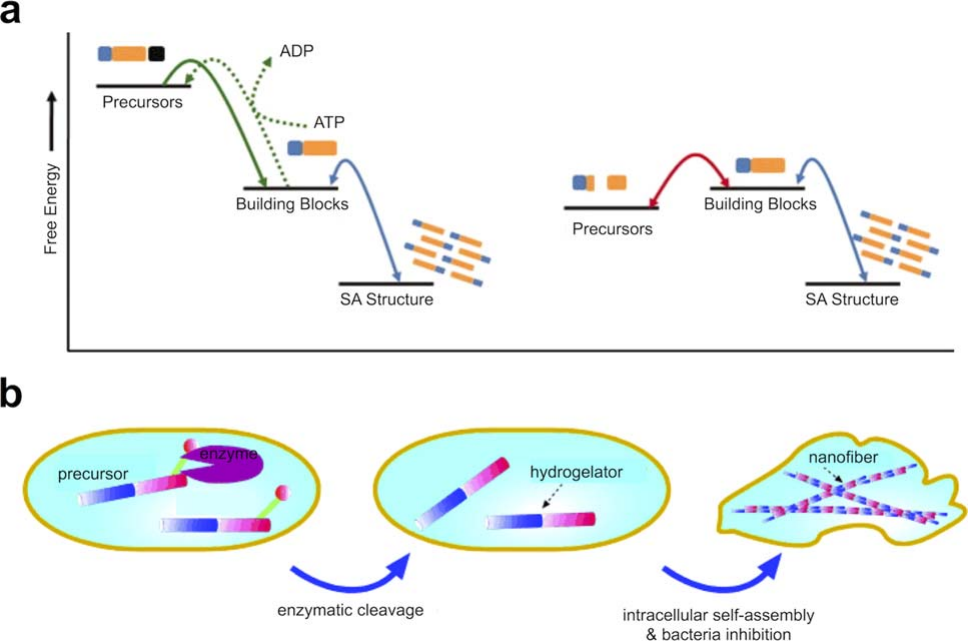
Examples showing methods for biologically regulated self‐assembly. (a) Free energy diagrams for two possible scenarios in enzyme‐assisted peptide self‐assembly. The first (left) illustrates a scenario in which the enzymatic reaction and self‐assembly process are both thermodynamically favored. In some cases, this scenario is reversible through the input of an energy source (i.e., ATP converted to ADP). The second free energy diagram (right) illustrates enzyme‐assisted self‐assembly under thermodynamic control, where the formation of a self‐assembling building block is thermodynamically unfavored in isolation, but yet may occur in a reversible way when coupled to sufficiently favorable self‐assembly. (b) An example illustrating intracellular formation of supramolecular peptide assemblies via activity of an intracellular enzyme, in this case a transformed enzyme in bacteria promotes conversion of a pro‐gelator molecule leading to intracellular assembly and cell arrest. Panel (a) reproduced with permission from Williams et al.^142^ Panel (b) reproduced with permission from Yang et al.^154^

In the context of “smart” disease therapeutics, biological indicators of disease are an especially relevant trigger to induce changes in materials. The most commonly used biological triggers are enzymes, which leverage biological energy sources (e.g., ATP) to chemically modify and/or structurally reorganize a material. Accordingly, a number of enzyme‐mediated drug delivery systems have been reported which use extracellular or intracellular enzymes to prompt release of a drug payload from a carrier.^74^ Supramolecular materials can similarly be designed to undergo chemical and/or structural changes as a result of the presence of enzymes. In particular, supramolecular materials based on peptide self‐assembly afford specific advantages since enzymatic substrates—which are often peptidic—can be incorporated within the primary sequence of a small molecule gelator.^139^, ^140^, ^141^ Enzymes can be used to convert a monomeric unit into an assembling motif (or vice versa) or to rupture bonds within the backbone of a peptide to reveal fragments that may then assemble or dissociate, depending on design of the system (Figure 4a).^142^ In an elegant example of this type of control, the use of the protease enzyme thermolysin, which can catalyze both peptide bond formation and hydrolysis, was demonstrated to facilitate peptide self‐assembly under thermodynamic control which is sensing and self‐correcting; furthermore the approach facilitates dynamic exchange of assembled species to amplify the most stable structures based on environmental conditions.^143^ In another route, taking advantage of the sequence specificity of certain matrix metalloproteinases (MMPs) results in the creation a pro‐gelator molecule that upon cleavage by MMP forms a molecule able to undergo supramolecular hydrogelation.^144^, ^145^ Sequences containing MMP cleavage sites could alternatively be used to stabilize a supramolecular assembly through covalent crosslinking, with the assembly becoming destabilized upon MMP‐cleavage of the crosslinking units and undergoing dissociation or structural changes.^146^ Nonpeptidic backbone units, such as esters, can also be incorporated within supramolecular assemblies and supramolecular polymers to facilitate enzyme‐responsive changes in the assembly or mechanical properties of the material.^147^ In another example, a hydrophilic antibiotic attached via a β‐lactam linkage to form a pro‐gelator peptide can be cleaved using β‐lactamase, releasing the drug and prompting assembly of the remaining peptide gelator.^148^ In a similar way, small molecule drugs can be tethered using enzyme‐degradable linkages for incorporation within supramolecular materials, for example, using ester linkages to release anti‐inflammatories.^149^

In addition to cleavage of backbone units within a supramolecular motif, other enzymatic approaches take advantage of the delicate interplay between attractive and repulsive forces in supramolecular peptide assemblies by leveraging more subtle chemical shifts to regulate or drive assembly. For example, the phosphorylation (using kinases) and dephosphorylation (using phosphatases) of R‐groups on serine and tyrosine residues has proven a useful, and in some cases reversible, trigger for controlling assembly of supramolecular peptide motifs.^19^ The ability to use enzymes to control supramolecular assembly affords many interesting and unique opportunities for producing “smart” therapeutics. Specific enzymes, such as those produced by cancer cells that act on amino acid side‐chain chemistry, can also be used to control supramolecular assembly.^140^ For example, cancer‐relevant enzymes can be used to induce a conformational change in nanostructure that results in release of an encapsulated chemotherapeutic,^141^, ^145^ or through catalytic induction of assembly from small molecule precursors to inhibit cell growth through promoting extracellular gelation.^150^, ^151^ Supramolecular peptide nanofibers may also contribute to cancer cell death by acting intracellularly, serving a prion‐like function in impeding cytoskeletal dynamics (Figure 4b).^152^, ^153^, ^154^ This phenomenon was further demonstrated to inhibit tumor growth in a concentration‐dependent manner when peptide nanostructures were injected into the vicinity of a tumor in an animal model.^155^

Although supramolecular peptide assemblies have been demonstrated to be the most amenable to enzymatic control of properties, other uses of enzymes to induce a structural and/or functional change in a supramolecular material have been demonstrated. For example, calix[*n*]arene‐capped mesoporous silica nanovalve particles have been designed to release encapsulated cargo upon enzyme‐mediated cleavage of esters or ureas incorporated within the backbone of a tethering strand.^156^ The incorporation of esters within the backbone of a binary supramolecular polymer prepared from terminal host‐guest interactions was also demonstrated to provide control over the assembled structure as a function of esterase content.^157^ A similar strategy has been used to prepare responsive vesicles from a fused supramolecular amphiphile that responds to the presence of esterases by rupturing and releasing encapsulated cargo.^158^, ^159^ Other polymeric micellar systems have been demonstrated where self‐assembly and geometry are a function of orthogonal enzyme triggers, and can thus be diversely regulated through incorporation of both a kinase/phosphatase switch along with a protease switch to afford multiple pathways of control in the resulting assembly.^160^

While enzymes have been the predominant biological route to impart supramolecular materials with responsive properties, other biologically relevant species could play a role in regulating assembly. As discussed previously, physiologically relevant small‐molecule redox agents can regulate the formation and function of supramolecular materials. Another biologically relevant route that has gained traction in recent years is the development of materials that can respond to changes in physiologic glucose concentrations.^161^ Although not typically thought of in the context of a supramolecular interaction, the binding of phenylboronic acids to glucose and similar diols forms a dynamic covalent bond with an affinity that can be tuned by external biological factors including pH and the presence of freely diffusible glucose to prepare materials that are dynamic, responsive, self‐healing, and capable of stimuli‐responsive release of an encapsulated cargo.^162^, ^163^ The equilibrium dynamics, reversibility, and specificity of this high‐affinity interaction are comparable to those observed in metal‐ligand coordination systems and thus it warrants consideration as a supramolecular motif with special utility in the context of preparing materials that can respond to a biologically relevant disease analyte such as glucose.

### 2.4 Mechanically responsive materials

Materials with tunable, controllable, and reversible mechanical properties could be broadly useful in the context of therapy, especially as an increasing body of knowledge continues to demonstrate the importance of mechanics, and furthermore dynamic mechanical properties, on the function of biological systems and relevance in disease.^164^, ^165^, ^166^, ^167^ Supramolecular materials offer specific advantages in that they can be easily tuned to modulate both mechanical and macroscopic properties as a result of rational design that leverages synergy and cooperatively between multiple noncovalent interactions. Individually, supramolecular interactions are inherently dynamic, exchanging between “bound” and “unbound” at a predictable rate. This knowledge contributes to a priori design through selection of particular supramolecular motifs to tune mechanical properties of the resulting material. Properties afforded by the dynamic and reversible character of supramolecular interactions also afford opportunity in the generation of shear‐thinning and self‐healing materials.^34^, ^57^, ^168^, ^169^, ^170^, ^171^, ^172^, ^173^ In contrast, materials prepared from traditional polymers are more likely to undergo mechanical loading and critical failure. Shear‐thinning and self‐healing properties would extend to minimally invasive routes for implantation via injection or catheter delivery, where the material restores its previous form once in the body. Tuning mechanical properties can additionally be exploited to modulate hydrogel erosion rate,^174^ an important feature to control drug or protein release that could also improve the responsiveness of a material to certain stimuli. The strength of supramolecular interactions, and their resulting effect on mechanical properties, can also be tuned to alter the biological compatibility and function in supramolecular materials.^64^, ^175^ Engineering of specific supramolecular interactions enables use of chemistries to post‐modulate mechanical properties, for example through secondary crosslinking, to provide additional control over mechanical properties at a later stage using an orthogonal mechanism.^88^, ^91^

**Figure 5:**
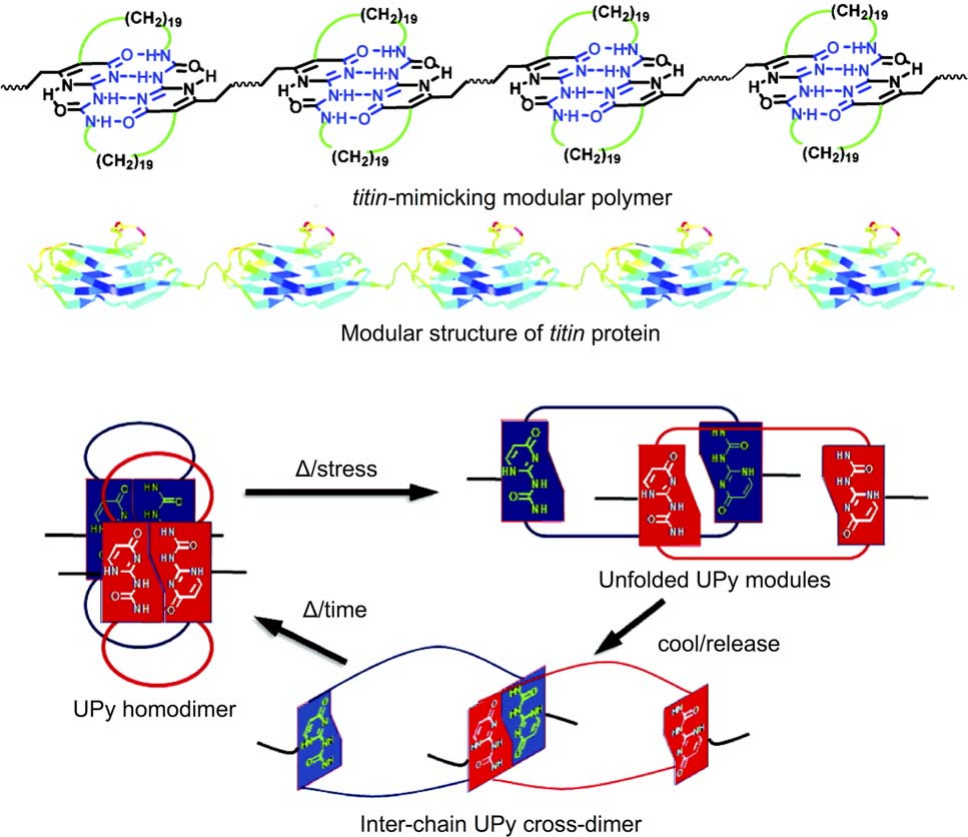
Example of a mechano‐stimuli‐responsive polymer prepared from supramolecular interactions between UPy motifs in conjunction with covalent bridging to produce a material that is tough and adaptive, mimicking the mechanism by which the *titin* protein in muscle responds to mechanical load. Figure reprinted with permission from Kushner et al.,^176^ Copyright 2009 American Chemical Society

While materials composed of dynamic and reversible bonds with “on/off” rates on the order of experimentally relevant timescales have inherent ability to respond to mechanical cues and reorganize or heal their structure, some supramolecular materials have been demonstrated with particularly elegant mechanisms to achieve mechano‐responsive properties. For example, a *titin*‐mimetic supramolecular material was demonstrated that recapitulates the properties of the native *titin* protein found in muscle in its high modulus, toughness, and elasticity, while simultaneously demonstrating adaptive and stimuli‐responsive properties (Figure 5).^176^ This polymer, prepared from a combination of UPy hydrogen bonding groups with covalent bridging between monomers, combines the reversible dynamics of the specific hydrogen bonding groups with the stability of a covalent bridge.

For the most part, the discussion of dynamic mechanical properties has focused on leveraging dynamics of interactions in equilibrium to confer tunable properties in the resulting materials. An interesting alternative approach has demonstrated mechanical properties of a supramolecular material that are governed by presence of a chemical fuel.^177^ In this work, transient nonequilibrium assemblies arise from energy derived from consumption of a fuel, leading to an unstable species that forms a mechanical hydrogel, which subsequently dissociates with hydrolysis of the gelator, restoring the precursor *sol* form. In a similar approach, the mechanical properties of a supramolecular material can be tuned through the presence of a catalyst, enabling acceleration of material formation and tuning of mechanical strength through a catalytically controlled process that accesses metastable assembly states.^178^ This overall strategy to tune the mechanics of a material through addition of an exogenous fuel or catalyst to access materials away from their equilibrium introduces a powerful method, perhaps uniquely reserved for supramolecular materials, to impart materials with responsive mechanical properties. Such an approach is highly biomimetic, as protein structures in nature use biological sources of energy or enzymatic help to access nonequilibrium and/or metastable structures. As such, the potential for such control over material properties to produce “smart” therapeutics could be great.

### 2.5 Controlling materials through affinity

Endowing materials with temporally tunable and controllable features has the possibility for great advantage in interacting with biological systems toward improved therapy, especially in light of the often‐dynamic nature of biology and disease pathology. In many of the strategies previously discussed here, supramolecular materials can be made responsive through the use of dynamic and specific interactions, and further the ability to use a stimulus to alter the energy landscape and change the state of the material in a reversible or unidirectional manner. However, even with a particular supramolecular motif, specific interactions are not created equal in affinity. This provides an opportunity to use gradated affinities in engineering controllable and responsive properties within materials, an advantage that may be uniquely possible using supramolecular design. One strategy would seek such control toward providing dynamic temporal control over bioactivity of a material. For example, decorating a material with a host macrocycle has been demonstrated as a strategy to alter bioactivity by “swapping” peptide‐based active sequences presented on the surface when these peptides are fused to hydrophobic guest molecules with differential affinity for the host.^179^ In this way, pro‐adhesive activity of a material in interacting with cells can be turned on and off. Materials incorporating host macrocycles can also be used in the generation of “refillable” drug delivery materials, as hydrophobic drug can be loaded remotely into the device at a high concentration and then be released over time at a rate dictated by binding equilibrium.^180^ A similar concept may be achievable using the precise binding and high affinity of oligonucleotide dimers.^181^

**Figure 6:**
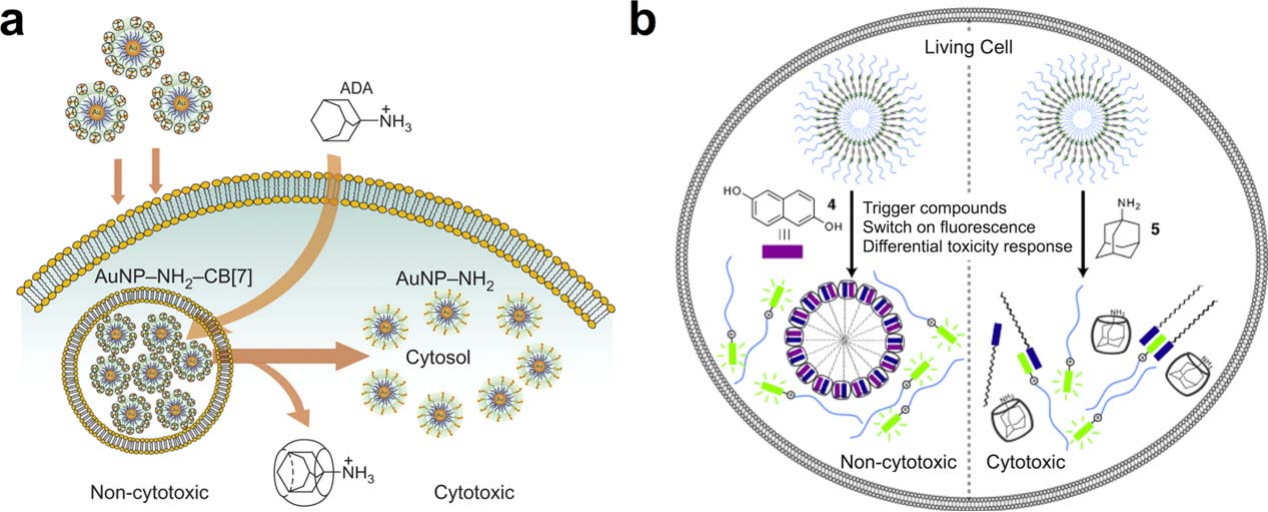
Examples illustrating responsiveness via modulating affinity. (a) A gold nanoparticle with a cationic shell screened using a supramolecular macrocycle (CB[7]) can be uncapped following internalization in a cell through addition of an adamantyl guest with higher affinity for the CB[7], leading to endosomal rupture and cytotoxicity. (b) A micellar assembly prepared from a building block fused by ternary complex formation within a macrocyclic host (CB[8]) can be disrupted through addition of a guest that competes with the ternary complex leading to fluorescence associated with disaggregation but not cell death, or alternatively a guest that disrupts the ternary complex formation leading to fluorescence and cytotoxicity. Panel (a) reprinted with permission from Macmillan Publishers Ltd: Nature Chemistry (Kim et al.^182^), Copyright 2010. Panel (b) reproduced with permission from Jiao et al.^183^

Affinity can also be used as a strategy to activate a therapeutic material by revealing hidden functionality. In one example, a gold nanoparticle surface‐modified with cationic amines can be capped with a cucurbit[7]uril macrocycle to screen the positive charge of the nanoparticle surface for uptake by cells; subsequent addition of a higher‐binding adamantyl species removes the macrocycle leading to an exposed cationic surface that is cytotoxic (Figure 6a).^182^ In a similar route, an amphiphilic building block prepared through ternary complex formation between a hydrophobic and hydrophilic block bridged by a macrocycle with two available sites for binding results in micellar assemblies that can be converted to fluorescent or cytotoxic materials upon selection of a freely diffusible competitive guest molecule (Figure 6b).^183^ In each of these examples, a cytocompatible nanoparticle is demonstrated that can be taken up by a cell and subsequently activated into a cytotoxic species through disruption of host‐guest interactions on the particle using competing guests with higher affinity. This type of remote‐activated therapy could be broadly applicable to a clinical setting whereby one could first ensure that a delivered material made it to the disease site of interest, and subsequently the therapy could be activated remotely through systemic delivery of the displacing guest molecule.

### 2.6 Multi‐stimuli‐responsive supramolecular materials

The preceding sections have taken a perhaps simplistic view when discussing the nature of stimuli used in the generation of responsive supramolecular materials. In truth, supramolecular materials can be engineered so as to respond to multiple stimuli. For example, materials that are inherently responsive to pH or ionic strength, such as supramolecular peptide assemblies, may also be engineered to incorporate light‐responsive chemistry or to present substrates for specific enzymatic reorganization, leveraging two independent mechanisms for stimuli‐responsiveness. Metallo‐supramolecular polymers have also been demonstrated that are independently thermo‐, photo‐, and chemo‐responsive.^92^ Multiple stimuli can alternatively be used to facilitate a dependent cascade response in a material, for example, by using a bulky photolabile group to inhibit the ability of a molecule to undergo temperature‐ or salt‐mediated association until the group has been cleaved. Finally, two stimuli can be combined cooperatively to control the equilibrium state of a small molecule gelator, such as was shown with biocatalytic control over supramolecular assembly state by the enzyme thermolysin in combination with light‐mediated control of assembly state using azobenzene.^184^ The versatility of supramolecular interactions, combined with rational bottom‐up design of these materials, thus lends itself to facile incorporation of multifarious components for sensing and responding to different stimuli.

## 3 Conclusions and therapeutic outlook

In this review, we have discussed a broad diversity of strategies to prepare responsive supramolecular materials with potential application toward the design of new therapeutics, specifically focusing on mechanisms of control that would have therapeutic and/or physiologic relevance. Strategies to engineer drug carriers with stimuli‐responsive control of properties may improve the specificity of therapy by providing a mechanism to sense disease state and/or disease site. Great potential has been demonstrated in the field of nanomedicine by using carriers, such as liposomes, to improve trafficking and limit off‐target effects of a drug toward improved efficacy and safety in pharmaceutical practice. Making such carriers “smart” marks a logical progression in achieving increased efficacy and safety through improving specificity of the therapy. Supramolecular design of materials uses organized noncovalent molecular recognition motifs in place of covalent bonds. Such interactions can be more easily overcome through chemical shifts that alter the thermodynamics of association and/or the kinetics of assembly. This affords extraordinary sensitivity in controlling the state and structure of a supramolecular assembly, and dramatic changes can therefore result from seemingly subtle alterations to the surrounding environment or by active and targeted manipulation using minimal external means. Efforts toward creating materials that might exhibit dynamic reciprocity with their biological environment through sensing a specific signal and responding by modulation of structural and functional properties remains a target of great interest, and one that may only be possible using dynamic noncovalent design rooted in supramolecular principles. As such, it is proposed here and elsewhere that supramolecular design principles provide several distinct advantages in preparing responsive drug carriers or implanted drug depots. Control over such systems may be manifest through the presence of markers of disease, or using a targeted stimuli; in either case the objective would be to control bioavailability of therapy at an appropriate dose, time, and place. Although efforts toward implementation of these principles have for the most part only begun, it is hoped that supramolecular engineering might contribute to superior therapeutic control rooted in rational molecular and material design, and this possibility warrants additional study.

Continued advancement in the coming years to develop new methodology in supramolecular chemistry may help to further improve the precision and sensitivity of supramolecular structures to realize increased control. In the context of drug delivery, improving the precision of supramolecular structures to realize discrete shape, size, and surface chemistry holds great promise, as these attributes dictate features such as biodistribution and pharmacokinetics when in circulation. Thus, variability in these traits could result in concomitant variability in therapeutic performance, raising issues in terms of both safety and efficacy. For example, many supramolecular materials used as drug carriers have a high aspect‐ratio geometry known to be beneficial in terms of enhancing circulation time.^185^ However, the dramatic polydispersity in the length of such assemblies that results from their typically open‐association pathway of formation and elongation may complicate efforts for predictable circulation properties. Strategies to control the growth or template the assembly of one‐dimensional supramolecular assemblies could therefore be useful in this regard.^186^, ^187^

In spite of the significant advantages that may be achieved through use of supramolecular engineering in the design of therapeutic materials, these approaches remain primarily unexplored in clinical use. A number of studies, highlighted here and elsewhere, have applied responsive supramolecular materials in the context of preclinical in vivo models of disease, lending support to the optimistic perspective provided in this review. However, until these strategies have been more thoroughly vetted by progressing through rigorous evaluation associated with a clinical trial, it is difficult to appreciate their possible impact. Some of the delay in implementation is attributable to the typical timeframe for adoption of new technologies in clinical practice. One must look no further than the field of nanomedicine to see that literally decades of preclinical work predate significant clinical implementation. However, this does not completely excuse the lack of supramolecular therapeutics in the clinic. In today's culture of pharmaceutical economics, the tremendous costs to bring a new therapy to market—often in excess of $1B USD—necessitate that any new therapy provide a very significant increase in performance while costs for development remain at an accessible level. The often complex chemistry required to create supramolecular materials can be multi‐step, low‐yielding, and cost‐prohibitive in this regard. As such, new routes to generate supramolecular materials must refine synthetic methodology and in many cases simplify molecular design. An additional barrier comes from a lack of familiarity for these systems in the context of research and development, business strategy, and regulatory approval. While the pathway for creating, evaluating, and implementing small molecule or antibody drugs has become more or less, routine, supramolecular materials introduce an array of new and often case‐specific challenges not encountered in the development of traditional drugs. This can be attributed, in large part, to the very specific molecular design of a supramolecular material that underlies its structure and function, meaning nothing in the development of these materials may ever become routine.

As outlined here, supramolecular design principles offer a number of advantages in preparing stimuli‐responsive materials. They could likewise have significant benefit in translation to clinical use. As such systems are typically modular, they could play a role in realizing the vision of personalized medicine and therapeutic customization. Additionally, many supramolecular materials are made from discrete building blocks, reducing the burden for assurance and characterization required compared to use of a polydispersed material building block. Such defined composition also would likely improve efforts to predict biodegradation products, thereby streamlining toxicology screening for materials and their degradation byproducts requisite in the early stages of clinical translation. For therapeutic impact to be realized, supramolecular materials must be designed for consistent application and reliable performance. This consideration becomes complicated when designing stimuli‐responsive materials, as their very function may require destabilizing and in some cases switching a system from the edge of an equilibrium or metastable state, a process that can be difficult to tune. Ultimately, one would want to ensure efficacy, safety, and predictable behavior for a stimuli‐responsive supramolecular material before broad therapeutic use can be realized.
